# Effects of Different Analysis Strategies on Paired Associative Stimulation. A Pooled Data Analysis from Three Research Labs

**DOI:** 10.1371/journal.pone.0154880

**Published:** 2016-05-04

**Authors:** Jacob Lahr, Sven Paßmann, Jonathan List, Werner Vach, Agnes Flöel, Stefan Klöppel

**Affiliations:** 1 Freiburg Brain Imaging, University Medical Center, Freiburg, Germany; 2 Department of Psychiatry and Psychotherapy, University Medical Center, Freiburg, Germany; 3 Department of Neurology, University Medical Center, Freiburg, Germany; 4 Department of Neurology, Charité Universitätsmedizin, Berlin, Germany; 5 Center for Medical Biometry and Medical Informatics, University of Freiburg, Freiburg, Germany; 6 Center for Stroke Research Berlin, Charité Universitätsmedizin, Berlin, Germany; 7 Cluster of Excellence NeuroCure, Charité Universitätsmedizin, Berlin, Germany; 8 Center of Geriatrics and Gerontology Freiburg, University Medical Center, Freiburg, Germany; University of Toronto, CANADA

## Abstract

Paired associative stimulation (PAS) is a widely used transcranial magnetic stimulation (TMS) paradigm to non-invasively induce synaptic plasticity in the human brain in vivo. Altered PAS-induced plasticity has been demonstrated for several diseases. However, researchers are faced with a high inter- and intra-subject variability of the PAS response. Here, we pooled original data from nine PAS studies from three centers and analyzed the combined dataset of 190 healthy subjects with regard to age dependency, the role of stimulation parameters and the effect of different statistical methods. We observed no main effect of the PAS intervention over all studies (F(2;362) = 0.44; p = 0.644). The rate of subjects showing the expected increase of motor evoked potential (MEP) amplitudes was 53%. The PAS effect differed significantly between studies as shown by a significant interaction effect (F(16;362) = 1.77; p = 0.034) but post-hoc testing did not reveal significant effects after correction for multiple tests. There was a trend toward increased variability of the PAS effect in older subjects. Acquisition parameters differed across studies but without systematically influencing changes in MEP-size. The use of post/baseline quotients systematically indicated stronger PAS effects than post/baseline difference or the logarithm of the post/baseline quotient. The non-significant PAS effects across studies and a wide range of responder rates between studies indicate a high variability of this method. We were thus not able to replicate findings from a previous meta-analysis showing robust effects of PAS. No pattern emerged regarding acquisition parameters that at this point could guide future studies to reduce variability and help increase response rate. For future studies, we propose to report the responder rate and recommend the use of the logarithmized post/baseline quotient for further analyses to better address the possibility that results are driven by few extreme cases.

## Introduction

Neuronal plasticity is the basis of learning and memory and leads to changes on a molecular, cellular and systemic level. On the synaptic level, long-term potentiation (LTP) and depression (LTD) are omnipresent mechanisms of neuronal plasticity. This bidirectional synaptic plasticity can be induced by tetanic stimulation at high (LTP) or low (LTD) frequencies or by associative pre- and postsynaptic stimulation and characteristically depends on the activity of postsynaptic NMDA receptors [1]. LTP/LTD have been studied extensively in animal tissue slices [2], and also in surgically removed human hippocampus specimens [3]. However, it cannot be assessed in the human brain in vivo.

Paired-associative stimulation (PAS) is one of the most frequently used transcranial magnetic stimulation (TMS) protocols to non-invasively induce neural plasticity in the intact human brain [4–6]. For PAS, electrical stimulation of a peripheral nerve (e.g. median nerve) and TMS of the contralateral primary motor cortex (M1) are repetitively coupled. Depending on the exact timing of the stimuli, this leads to an increase or decrease of motor evoked potentials (MEP). At an interstimulus-interval of 25 ms, MEP amplitudes are increased, and at an interstimulus interval of 10 ms, the amplitudes are decreased [5]. MEP amplitude changes have been shown to begin directly after PAS intervention and to last for a duration of at least one hour [4,6].

Experimental paradigms that induce neural plasticity in the intact human brain are often referred to as „LTP-like”as they share some but not all characteristics with LTP and as the underlying mechanisms are not completely understood. For PAS, three requirements for an LTP-like mechanism, namely stimulus-timing dependence [5], NMDA-receptor dependence [7] and associativity [4] have been demonstrated.

A large scale quantitative review provided strong indication for a robust PAS effect which was strongest for an inter-trial interval of 5 or 20 s [6]. On the other hand, a high inter-individual variability and a response to PAS in the expected direction in only 60% and even 39% of participants found in two well powered studies challenge such conclusions [8,9]. Small effect sizes and a high variability have recently been reported not only for PAS but also for other methods of non-invasive brain stimulation and are intensively debated [10,11]. Despite that, systematic alterations of PAS have been demonstrated in a variety of neuropsychiatric disorders, including Alzheimer’s disease [12,13], depression [14], schizophrenia [15] or writer’s cramp [16].

Previous studies identified several factors such as age [8], time of day [17], attention [18] or cortical anatomy [19] to account for some of its variability, but these were not necessarily replicated and even small changes in the parameter sets may impact the results substantially. There is not yet a consensus if optical navigation for TMS coil placement has a positive effect on the variability of MEPs [20–22]. Other factors include the strength of the peripheral electrical stimulation which affects the number of stimulated afferent fibers as well as the strength of the cortical stimulation as this will influence the number of I-waves [23]. One study [8] found that a lower stimulator output necessary to induce a 1mV MEP correlated with a stronger PAS effect.

Finally, the choice of data analysis methods and statistical analyses also influences the results. This concerns the averaging across the individual trials of a participant at a given time point (e.g. at baseline or at various intervals after the PAS-intervention) and the averaging across individuals for each time point but also the transformations (post/baseline differences, quotients, or logarithmized quotients). The majority of studies relies on post/baseline differences for analysis of the main PAS effect by using ANOVA, and on quotients for further analyses such as e.g. correlations.

Our own experience [24–30] and that of others [8,9] with PAS but also relatively well powered studies using other means for non-invasive brain stimulation [19,31] led us to perform a meta-analysis on original data for comparison to the recent review by Wischnewski and colleagues [6]. These authors reported that the PAS protocol remained robust even after removing studies with potential overestimation of effect size. On the other hand, their analysis had to be based on published data while ample evidence indicate that studies with a null effect tend to remain unpublished [32].

We therefore performed a meta-analysis based on our original published and unpublished data from nine studies performed by two TMS research groups at three different laboratories. We aimed at evaluating the robustness of the PAS effect. In addition, we sought to quantify the influence of stimulation parameters, age, gender and statistical methods in the analysis of PAS. We additionally tested for associations between the extend of the PAS effect and age as well as magnetic stimulation strength as both has been indicated in a previous study [8]. Finally, we also examined correlations between PAS and the peripheral electrical stimulation strength.

## Methods

### Subjects

Healthy control subjects from nine studies conducted at three German centers (Charité Universitätsmedizin Berlin, University Medical Center Münster, University Medical Center Freiburg) were included in this study (Table 1). The studies were approved by the respective local ethics commissions (Ethik-Kommission der Albert-Ludwigs-Universität Freiburg for studies A & B, Ethik-Kommission der Ärztekammer Westfalen-Lippe und der Westfälischen Wilhelms-Universität Münster for studies C & D, Ethikkommission der Charité Universitätsmedizin Berlin for Studies E—I) and all participants gave their written informed consent.

**Table 1:** Summary of subjects and studies.

study	center	age (years)	gender (f/m)	N	Responder rate	initially published in
A	Freiburg	69.6 ± 5.7	19/9	28	53.6% (15)	Lahr et al. [30]
B	Freiburg	24.0 ± 2.0	14/18	32	62.5% (20)	Klöppel et al. [29]
C	Münster	49.9 ± 8.3	8/4	12	16.7% (2)	List et al. [24]
D	Münster	70.5 ± 3.6	10/10	20	80% (16)	List et al. [26]
E	Berlin	63.6 ± 12.8	4/6	10	60% (6)	List et al. [28]
F	Berlin	65.3 ± 5.2	12/8	20	35% (7)	Unpublished
G	Berlin	63.9 ± 6.2	16/14	30	60% (18)	List et al. [27]
H	Berlin	25.8 ± 5.9	2/21	23	52.2% (12)	Unpublished
I	Berlin	64.3 ± 6.1	7/8	15	33.3% (5)	Unpublished
∑			92/98	190		

### PAS acquisition protocols

The differences of the experimental procedures of the included studies are outlined in Table 2.

**Table 2:** Methodological differences in the PAS paradigm between the studies.

Study	A & B	C & D	E	F	G	H	I
PAS	180 paired stimuli, interval of 5 s, interstimulus interval of 25 ms between electrical and magnetic stimulus	90 paired stimuli, interval of 20 s, ISI 25 ms between electrical and magnetic stimulus	132 paired stimuli, interval of 5 s, ISI 25 ms between electrical and magnetic stimulus	132 paired stimuli, interval of 5 s, ISI 25 ms between electrical and magnetic stimulus	132 paired stimuli, interval of 5 s, ISI 25 ms between electrical and magnetic stimulus	132 paired stimuli, interval of 5 s, ISI 25 ms between electrical and magnetic stimulus	132 paired stimuli, interval of 5 s, ISI 25 ms between electrical and magnetic stimulus
TMS	TMS adjusted to 1 mV unconditioned MEP amplitude.20 MEPs per condition. Post measurements at 0, 8 and 15 minutes post intervention.	TMS 0.5–1.0 mV interval 10 s, 20 MEPs per condition. Post measurements at 0, 15, 30 (60 min for study C) post intervention.	TMS 0.5–1.0 mV; 10 MEPs per condition; post measurements at 0, 15, 30 post intervention.	TMS 0.5–1.0 mV; 10 MEPs per condition; post measurements at 0, 15, 30 post intervention.	TMS 0.5–1.0 mV; 10 MEPs per condition; post measurements at 0, 15, 30 post intervention.	TMS 0.5–1.0 mV; 10 MEPs per condition; post measurements at 0, 15, 30 post intervention.	TMS 0.5–1.0 mV; 10 MEPs per condition; post measurements at 0, 15, 30 post intervention.
E-Stim	300% of perception threshold at median nerve	300% perception threshold at ulnar nerve	300% of perception threshold at median nerve	300% perception threshold at median nerve (some participants did not tolerate stimulation at 300% perception threshold, there the stimulation intensity was adjusted individually (mean 273%), a visible twitch of the thumb was required)	300% of perception threshold at median nerve	300% of perception threshold at median nerve	300% perception threshold at median nerve (some participants did not tolerate stimulation at 300% perception threshold, there the stimulation intensity was adjusted individually (mean 277%), a visible twitch of the thumb was required)
Muscle	APB (right Hand, only right handed subjects)	ADM (dominant hand)	APB (right Hand, only right handed subjects)	APB (right Hand, only right handed subjects)	APB (right Hand, only right handed subjects)	APB (right Hand, only right handed subjects)	APB (right Hand, only right handed subjects)
Other	Neuronavigation, Attention monitored by counting visual stimuli Experiment conducted in the afternoon.	Experiment conducted between 10 AM and 3 PM Attention monitored by counting number of ulnar nerve stimulations		Experiment conducted between 9 AM and 6 PM Attention monitored by counting number of ulnar nerve stimulations		Experiment conducted between 9 AM and 6 PM Attention monitored by counting number of median nerve stimulations	Experiment conducted between 9 AM and 5 PM Attention monitored by counting number of ulnar nerve stimulations

### Data Processing and Statistical Analysis

The mean MEP size was calculated for each time-point (baseline and post-measurements) and for each subject. Testing MEPs for normality using Shapiro-Wilks test indicated no normal distribution. A repeated measures analysis of variance (rmANOVA) was calculated using SPSS software (Version 22.0) with the factor TIME (three levels: before PAS (pre), directly after PAS (post0) and 15 min after PAS (post15) as these measurements were available in all studies) as repeated measures factor and STUDY (nine levels) as between subjects factor. In case of a significant interaction, Games-Howell correction was applied to post-hoc testing. Degrees of freedom were adjusted by the Huynh-Feldt method, when the assumption of sphericity was violated.

Three different data transformations of the baseline and averaged post MEP measurements were compared: the difference between post and baseline (PAS_diff_), the post/baseline quotient (PAS_quot_), and the logarithm of the quotient (PAS_logquot_).

A random-effects regression model was fitted to each of the transformed data sets using the metafor library in R [33] and heterogeneity between studies was assessed with Cochrane’s Q-test. Results are displayed using a Forest plot.

For calculation of the responder-rate, the quotient of the averaged post measurements and the baseline measurement effect was calculated [8]. Subjects attaining values above one were thus considered as PAS-responders. Rank based correlation (Spearman’s rho) between age, TMS intensity (in percent of maximal stimulator output: %MSO), electrical peripheral nerve stimulation intensity (mA), PAS_logquot_ and |PAS_logquot_| was calculated. Following the approach by Müller Dahlhaus et al. (2008) association between age and the absolute variability (i.e. MEP increases or decreases) induced by PAS was assessed by correlating |PAS_logquot_| with age using Spearmans’s rho. Association between gender and PAS_logquot_ and between inter-trial interval (5 s or 20 s) and PAS_logquot_ were assessed using point-biserial correlation which is equivalent to a t-test but directly provides a measure of effect size.

To assess the influence of different data transformations, we visualized MEPs relative to baseline from all participants displaying the mean from all participants of a given timepoint and study. This visualization was contrasted with a visualization where the mean and the standard error were calculated on the logarithmized data. To further underline the influence of different averaging methods, we also visualized the mean, the median, and the mean of the log-transformed data that was back transformed to linear space using the following formula:

$${log\;transformed\;data=\;10}^{mean(log10(data))}$$

## Results

Data from PAS experiments of nine different studies were analyzed using an rmANOVA on the baseline, post0, and post15 measurements (Tables 1 and 2). There was no main effect of TIME over all studies (F(2;362) = 0.44; p = 0.644; Table 3) while the main effect of STUDY was significant (F(8;181) = 2.04; p = 0.044; Table 3). The overall responder rate was 53.2% (101 out of 190 subjects). The interaction TIME x STUDY was significant (F(16;362) = 1.77; p = 0.034; Table 3) but post-hoc testing did not reveal significant effects after correction for multiple testing (Games-Howell procedure; minimal p-value = 0.256). Responder rates for the individual studies were between 16.7–80% (Table 1).

**Table 3:** PAS effect over time. Results from rmANOVA over all studies (measurements at baseline, post0 and post15). Bold letters indicate significant effects.

Study	Term	F (df)	P	responder rate
All studies	TIME	0.44 (2;362)	0.644	53.2% (101/190)
All studies	STUDY	2.04 (1; 181)	**0.044**	
All studies	TIME x STUDY	1.77 (16;362)	**0.034**	

**Fig 1:**
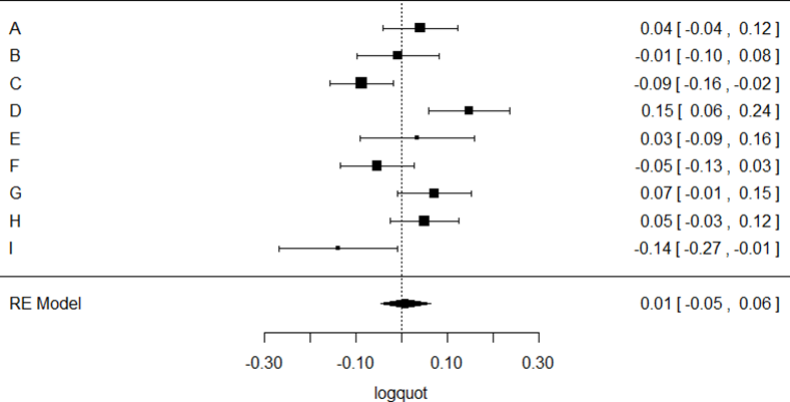
Forest plot of PAS-effect calculated with the logarithmized MEP ratio between the averaged post measurements and baseline (PAS_logquot_). The right column lists the corresponding mean and 95% confidence interval for the individual studies, below the estimated effect across all studies is indicated.

None of the three random-effects meta-analyses indicated an overall effect of PAS. The model based on the post/pre quotients indicated a (not significantly) higher effect of PAS (PAS_quot_: 1.11 ± 0.08; S1 Fig), while the models based on the differences (PAS_diff_: 0.02 ± 0.06 mv; S2 Fig) and logarithmized quotients (PAS_logquot_: 0.01 ± 0.03; Fig 1) indicated comparable results. Heterogeneity as assessed by Cochrane’s Q was significant at a level p<0.001 for all three models.

**Fig 2:**
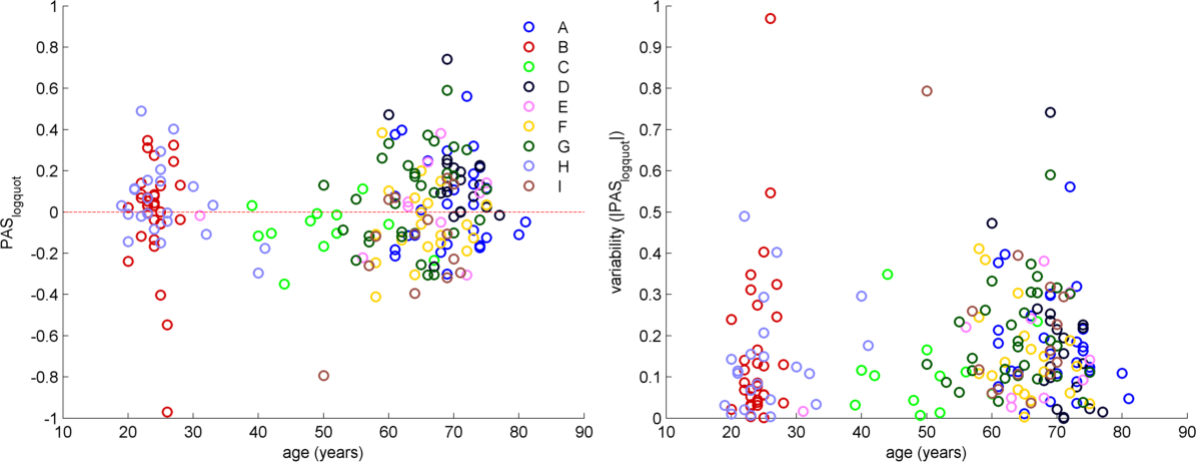
Association between PAS_logquot_ (left panel), variability induced by PAS_logquot_ (|PAS_logquot_|, right panel) and age. Letters indicate separate studies (see Table 1).

**Fig 3:**
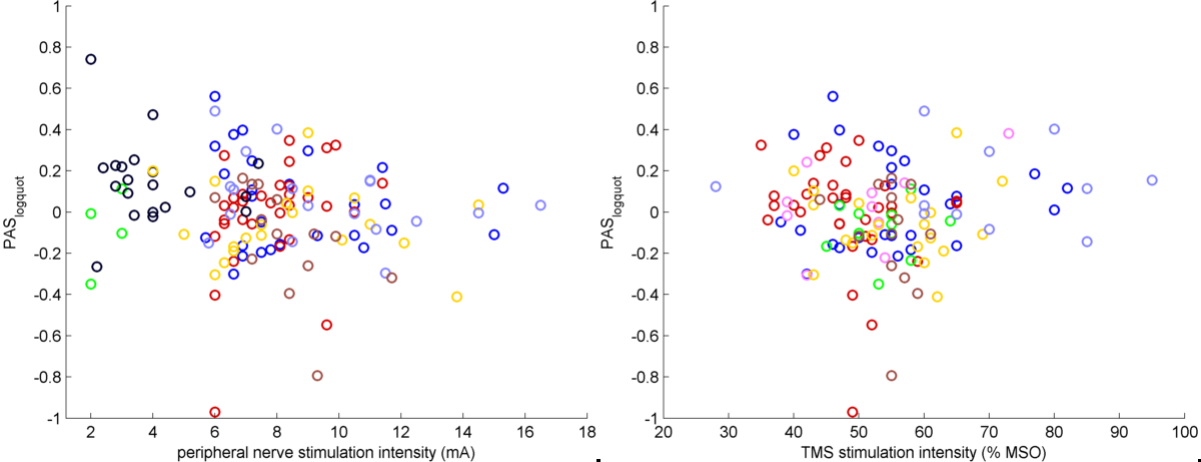
Scatter plot displaying the association between PAS_logquot_ and the intensities of peripheral nerve stimulation (left panel) and TMS (right panel). MSO: maximum stimulator output.

There was a trend towards a positive association between variability of PAS_logquot_ and age (rho = 0.13; p = 0.068; Fig 2), and no significant correlation between PAS_logquot_ and age (rho = 0.07; p = 0.308; Fig 2), electrical peripheral nerve stimulation intensity (rho = -0.15; p = 0.091, Fig 3, left panel) and TMS intensity (rho = -0.01; p = 0.897, Fig 3, right panel) or variability of PAS_logquot_ and electrical peripheral nerve stimulation intensity (rho = -0.01; p = 0.139) and TMS intensity (rho = 0.04; p = 0.689). There was also no significant association between PAS_logquot_ and gender (r = 0.01; p = 0.851), the use of neuronavigation (r = 0.01; p = 0.923) or the inter-trial interval during the PAS intervention (5 s vs. 20 s; r = 0.09; p = 0.239).

**Fig 4:**
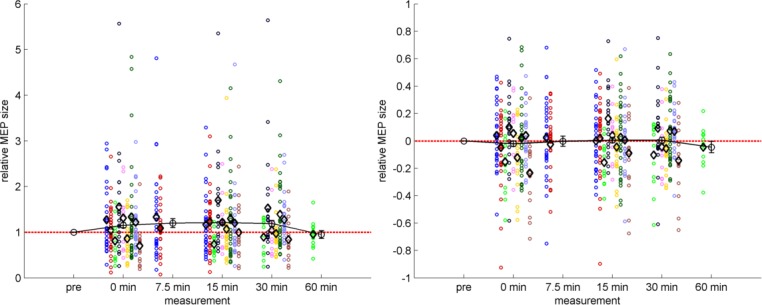
PAS-induced effects. Mean MEP amplitudes following PAS are shown relative to the baseline level. Each study is represented by a distinct color as in Figs 2 and 3, each circle represents the measurements of one subject, and each black diamond marker represents the mean of a study at the given time-point. The black line with error bars represents the temporal course of PAS across subjects and studies. The dashed red line represents no change against the baseline measurement. Left panel: normalized data ± SEM. Right panel: same PAS data but log-transformed prior to calculation of means and SEM. Note that the x-axis depicts distinct time points rather than a continuous scale.

The left panel of Fig 4 displays the PAS effect relative to baseline with means and SEM calculated across all studies indicating relatively high MEP values and a strong effect of outliers. In contrast, data in the right panel were log-transformed before calculating the means and SEM.

**Fig 5:**
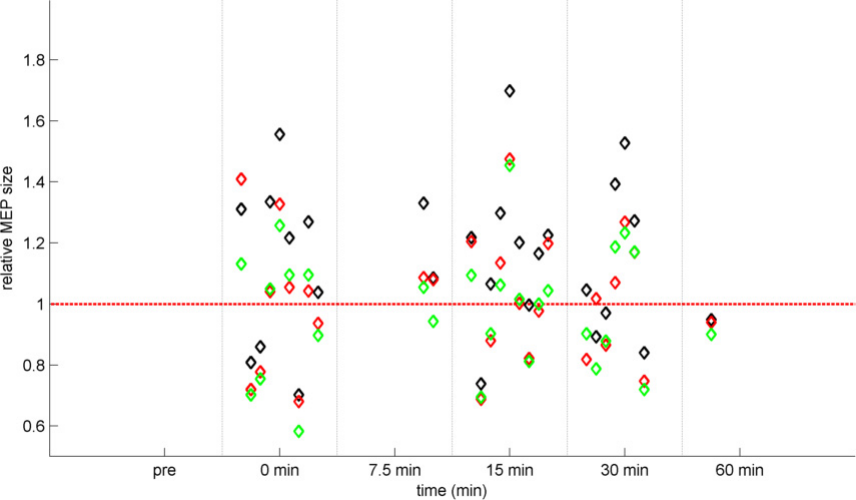
Comparison of methods to calculate the average PAS response. Each column of diamond markers represents the different averages of one study: the mean (black), the median (red), and the mean as calculated on the log-transformed data that was back-transformed to linear space (green). The mean values are systematically higher than those of the median or those of the mean calculated on the log-transformed data.

The influence of different methods to estimate the average PAS response was further assessed by comparing the mean of the raw data to the median and the back-transformed mean of the logarithmized data. The mean yielded the highest values in 17 out of 19 cases (Fig 5).

## Discussion

Integrating original data from almost 200 subjects, we did not observe a significant effect of PAS (i.e. TIME) but found that the PAS effect differed significantly between studies. However, post-hoc testing did not reveal differences between individual studies after correction for multiple comparisons. The wide range of responder rates together with the differences of the PAS effect between studies argue for a high variability. The results of our study are therefore different from a recent quantitative review showing robust PAS effects across all examined post PAS intervals [6]. Although that study did not access original data from individual subjects, they integrated data from 60 individual studies and made an effort to ensure that results of their meta-analyses were not driven by a number of small scale studies that would report unrealistically large effect-sizes.

Our data were acquired at three sites by two TMS research groups. Although both teams were working independently from each other at the time of data acquisition, we cannot fully exclude the possibility that both teams performed the PAS intervention incorrectly. However, well powered single studies by other research groups with ample experience in neurophysiology [8,9] also found no evidence for a robust PAS effect, and overall low responder rates.

In line with previous findings [8], we found no association between age and the strength of the PAS effect. In contrast to that study, we did not find a smaller but a higher variability of the MEP after PAS with increasing age. Of note, we did not examine a continuous sample across the whole age range and especially middle aged subjects (i.e. 30–50 years old) are underrepresented in the study sample. Between-study differences could therefore influence age effects. There was also no correlation between the strength of the PAS effect and gender, in line with recent work [34]. In additional correlation analyses we examined the effect of the strength of the electrical and magnetic stimulation on the PAS effect and found no significant correlation, in contrast to [8] who reported stronger PAS effects in those requiring a lower stimulator strength to induce a 1 mV MEP.

We were unable to evaluate the effect of different TMS vendors which had recently been suggested [35] as all studies used the same system. In addition, we did not find a systematic difference between studies with and without a navigation system, findings which add to an ongoing and so far inconclusive debate [20–22].

Importantly, we were able to demonstrate in this meta-analysis that the choice of statistical analysis has a distinct impact on the results: MEPs are not normally distributed and, furthermore, are by definition positive, implying that outliers systematically lead to an overestimation of the mean (e.g. single MEP measurements can be above 3 mV, but never below 0 mV). This effect is further magnified when parametric statistics are applied to MEP values relative to a baseline measurement as the baseline measurement takes place directly after adjusting stimulation parameters and coil position to acquire stable MEPs with low variability. Small movements or fluctuations of attention may thus have a higher impact on the subsequent post-measurements. Even if there is no external gold standard to validate the statistical method, the positive skewness of the data leads us to propose to log-transform the post/baseline quotient for further statistical processing as this leads to a more normal data distribution and to use absolute MEP values (baseline- and post-measurements) for a rmANOVA. Although not the focus of this study, the same arguments can also be applied to data from individual trials for a given subject and time point where either mean or median can be used to average across trials and data may already be log-transformed at this stage.

In summary, we demonstrate a high variability of the PAS-protocol leading to an overall non-significant effect of the intervention. Given this high variability, PAS-results in neurological and psychiatric patients should be interpreted with precaution. Conclusions drawn from single subject experiments do not yet seem to be reliable, and studies with higher patient numbers are needed to prove the validity of this paradigm in a clinical context. Detailed description of acquisition parameters, blinding the subject and the examiner to group status and even stimulation protocol (e.g. by involving a second experimenter who switches between excitatory 25 ms and inhibitory 10 ms intervals), carefully controlling for potential confounders such as age, gender, attention and a statistical analysis plan robust against outliers seem the best approach to handle PAS data. Moreover, future should investigate further sources of intra- and intersubject variability as it may have a tractable physiological underpinning. A recent approach to track down the high variability of noninvasive brain stimulation paradigms is using brain-state dependent TMS stimulation and thus adjusting the timing of TMS stimulation by real-time analysis of EEG [36,37]. Brain-state dependent variability of PAS may not only explain discrepancies between PAS studies, but also a high intrasubject variability of PAS [38].

## Supporting Information

S1 Fig Forest plot of PAS-effect calculated with the MEP ratio between the averaged post measurements and baseline (PAS_quot_). The right column lists the corresponding mean and 95% confidence interval for the individual studies, below the estimated effect across all studies is indicated.
(TIF)

S2 Fig Forest plot of PAS-effect calculated with the MEP difference between the averaged post measurements and baseline (PAS_diff_). The right column lists the corresponding mean and 95% confidence interval for the individual studies, below the estimated effect across all studies is indicated.
(TIF)
