# Supplementary figures and images for: Effects of Different Analysis Strategies on Paired Associative Stimulation. A Pooled Data Analysis from Three Research Labs
Source: PLoS One. 2016 May 4;11(5):e0154880. doi: 10.1371/journal.pone.0154880 (PMC4856316; doi:10.1371/journal.pone.0154880)

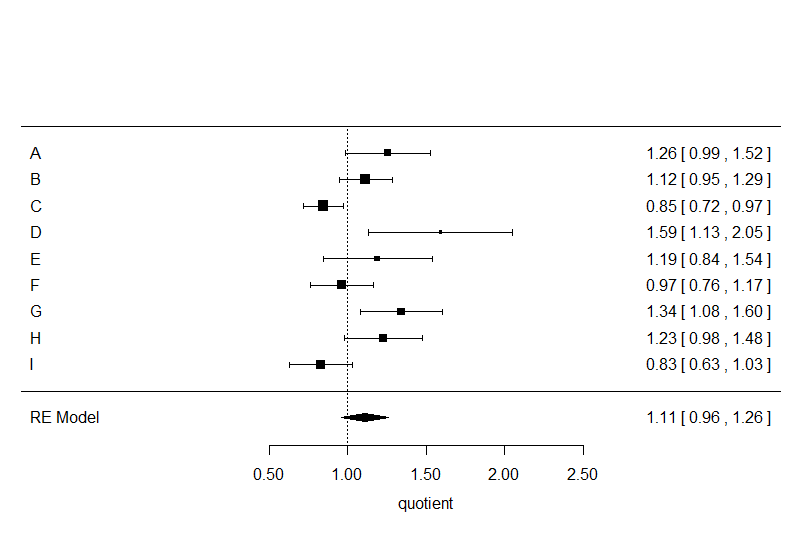

Supplement: S1 Fig — The right column lists the corresponding mean and 95% confidence interval for the individual studies, below the estimated effect across all studies is indicated. (TIF) [file pone.0154880.s001.tif]

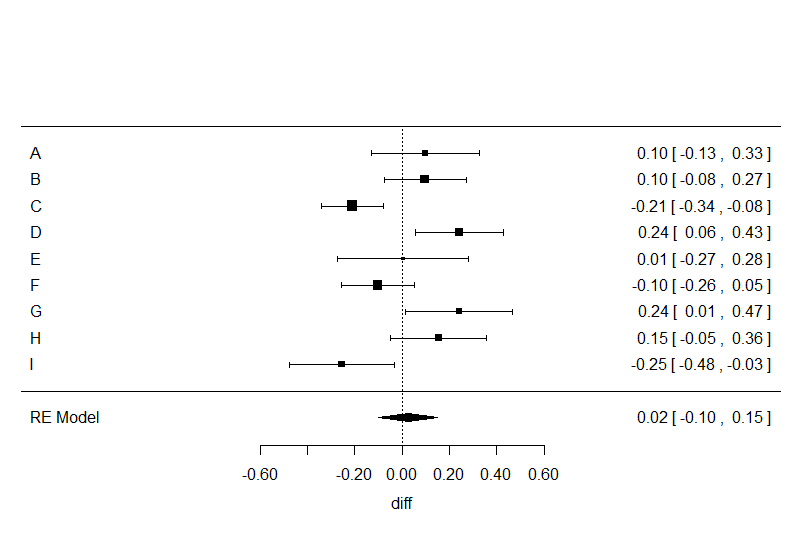

Supplement: S2 Fig — The right column lists the corresponding mean and 95% confidence interval for the individual studies, below the estimated effect across all studies is indicated. (TIF) [file pone.0154880.s002.tif]
